# Understanding the Influence of Crop Residue Burning on PM_2.5_ and PM_10_ Concentrations in China from 2013 to 2017 Using MODIS Data

**DOI:** 10.3390/ijerph15071504

**Published:** 2018-07-17

**Authors:** Yan Zhuang, Danlu Chen, Ruiyuan Li, Ziyue Chen, Jun Cai, Bin He, Bingbo Gao, Nianliang Cheng, Yueni Huang

**Affiliations:** 1 State Key Laboratory of Earth Surface Processes and Resource Ecology, College of Global Change and Earth System Science, Beijing Normal University, 19 Xinjiekouwai Street, Haidian, Beijing 100875, China; yzhuang@mail.bnu.edu.cn (Y.Z.); dlchen@mail.bnu.edu.cn (D.C.); leeruiyuan@bjfu.edu.cn (R.L.); hebin@bnu.edu.cn (B.H.); 2 Joint Center for Global Change Studies, Beijing 100875, China; 3 Department of Earth System Science, Tsinghua University, Beijing 100084, China; cai-j12@mails.tsinghua.edu.cn; 4 National Engineering Research Center for Information Technology in Agriculture, 11 Shuguang Huayuan Middle Road, Beijing 100097, China; gaobb@nercita.org.cn; 5 College of Water Sciences, Beijing Normal University, 19 Xinjiekouwai Street, Haidian, Beijing 100875, China; 15001195306@163.com; 6 Department of Physics, Beijing Normal University, 19 Xinjiekouwai Street, Haidian, Beijing 100875, China; huangyueni@mail.bnu.edu.cn

**Keywords:** PM concentrations, crop residue burning, correlation analysis, interannual and seasonal variations, China

## Abstract

In recent years, particulate matter (PM) pollution has increasingly affected public life and health. Therefore, crop residue burning, as a significant source of PM pollution in China, should be effectively controlled. This study attempts to understand variations and characteristics of PM_10_ and PM_2.5_ concentrations and discuss correlations between the variation of PM concentrations and crop residue burning using ground observation and Moderate Resolution Imaging Spectroradiometer (MODIS) data. The results revealed that the overall PM concentration in China from 2013 to 2017 was in a downward tendency with regional variations. Correlation analysis demonstrated that the PM_10_ concentration was more closely related to crop residue burning than the PM_2.5_ concentration. From a spatial perspective, the strongest correlation between PM concentration and crop residue burning existed in Northeast China (NEC). From a temporal perspective, the strongest correlation usually appeared in autumn for most regions. The total amount of crop residue burning spots in autumn was relatively large, and NEC was the region with the most intense crop residue burning in China. We compared the correlation between PM concentrations and crop residue burning at inter-annual and seasonal scales, and during burning-concentrated periods. We found that correlations between PM concentrations and crop residue burning increased significantly with the narrowing temporal scales and was the strongest during burning-concentrated periods, indicating that intense crop residue burning leads to instant deterioration of PM concentrations. The methodology and findings from this study provide meaningful reference for better understanding the influence of crop residue burning on PM pollution across China.

## 1. Introduction

Recently, particulate matter (PM) pollution has become a hot spot concerning people’s life and health [1,2,3]. Both PM_10_ (coarse particles with aerodynamic diameter between 2.5 μm and 10 μm) and PM_2.5_ (fine particles with aerodynamic diameter equal to or less than 2.5 μm) have been considered as major air pollutants in China [4]. A great deal of research [5,6,7] has proved that in addition to haze-induced low visibility, sustained exposure to high concentrations of PM_10_ and PM_2.5_ is harmful for human’s physical and mental health. On the other hand, short-term exposure or low-concentration exposure also adversely affects corporeity or even birth outcomes [8,9,10]. Furthermore, the morbidity of respiratory disease, cardiovascular disease, and lung cancer are strongly correlated with severe PM_2.5_ pollution [11]. However, although the government has taken some effective emission-reduction measures to alleviate the air pollution, PM concentrations still significantly exceed the guideline value proposed by the World Health Organization (WHO) in many cities of China [12]. There are two major drivers for the PM pollution, anthropogenic activities, and unfavorable meteorological conditions [13,14,15]. With increasing anthropogenic emission, PM pollution is hard to ameliorate in a short time [16]. Specifically, biomass burning and secondary pollutant formation are two main sources for PM pollution in China [17]. 

Crop residue burning, as one type of biomass burning, is a convenient, yet less environmentally friendly way to dispose massive agricultural wastes. For China, agricultural production plays an important role in the national economy, which means a large number of crop residues, such as paddy straws, wheat straws, and corn stalks, are generated and piled up on bare croplands. Following this, substantial crop residues are burnt directly to fertilize the soil and prepare for next crop-planting season. However, the burning of crop residues has seriously influenced the local and regional air quality during harvest seasons, especially in Northeast China [18,19,20]. During the burning process, severe haze episodes are further aggravated because SO_2_ and NO_X_ can be oxidized into secondary inorganic/organic aerosol (SIA/SOA), which are important sources for generating secondary PM_2.5_ [17]. In addition, other aerosol emissions from crop residue burning result in the decline of local precipitation to a certain extent, leading to the further increase of PM_2.5_ concentrations [21,22]. In other words, the change of meteorological conditions caused by crop residue burning may further exacerbate PM pollution. Therefore, in order to mitigate the current ambient air pollution, it is highly urgent to take effective and targeted measures to control crop residue burning in China.

Due to the vast territory of China, PM concentrations and the condition of crop residue burning demonstrate notable temporal and spatial difference across China. Given the potential risk PM exert on public health, it is essential to explore correlations between crop residue burning and PM concentrations. Yin et al. revealed the spatial distribution of crop residue burning and PM_2.5_ concentrations in China at a seasonal pattern [23], and Chen et al. discussed the influence of crop residue burning on PM_2.5_ concentration in Heilongjiang Province of China during a severe haze episode [24]. Zhuang et al. analyzed the trend of crop residue burning in different regions of China from 2003 to 2017 [18]. Meanwhile, some related studies have been conducted in other countries, such as India and Thailand. Awasthi et al. explored the effect of crop residue burning on pulmonary function tests of youth in North West India [25]. Although many scholars [26,27,28] have discussed the emissions from crop residue burning, limited studies have been conducted on understanding correlations between crop residue burning and PM concentrations. To fill this gap, we attempt to understand the spatio-temporal variation of PM concentrations across China and its correlation with crop residue burning. Firstly, from a regional perspective, we conducted spatio-temporal trend analyses of PM (including PM_2.5_ and PM_10_) concentrations in China during 2013 to 2017. Next, we analyzed interannual and seasonal variations of crop residue burning in different regions across China. Following this, we analyzed the correlation between PM concentrations and crop residue burning in different regions at different temporal scales.

## 2. Materials and Methods

### 2.1. Study Area

**Figure 1:**
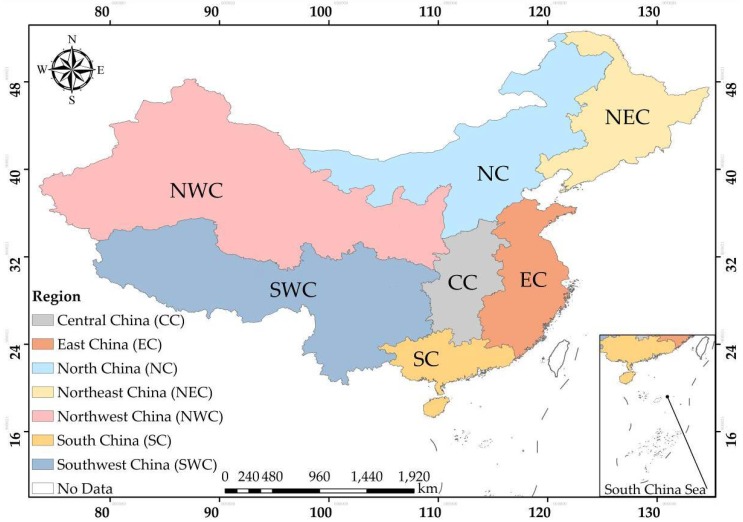
Geographical locations of seven regions in China.

China has a vast territory area of about 9.6 million square kilometers with complicated terrain and climatic characteristics in different regions, which leads to different-periods of agricultural crops-planting and reaping. Considering the notable spatio-temporal patterns of crop residue burning in China, we divided the study area into seven regions (Figure 1) according to Chinese administrative divisions [29]. The seven regions are named as follows: Northeast China (NEC, including Heilongjiang Province, Jilin Province, Liaoning Province), North China (NC, including The Inner Mongolia Autonomous Region, Shanxi Province, Hebei Province, Beijing, Tianjin), Northwest China (NWC, including Shaanxi Province, Gansu Province, The Ningxia Hui Autonomous Region, Qinghai Province, The Xinjiang Uygur Autonomous Region), East China (EC, including Shandong Province, Jiangsu Province, Zhejiang Province, Fujian Province, Anhui Province, Jiangxi Province), Central China (CC, including Henan Province, Hubei Province, Hunan Province), South China (SC, including Guangdong Province, The Guangxi Zhuang Autonomous Region, Hainan Province), and Southwest China (SWC, including Sichuan Province, Guizhou Province, Yunnan Province, The Tibet Autonomous Region).

### 2.2. Data Sources

#### 2.2.1. Ground-Observed PM_2.5_ and PM_10_ Concentrations Data

**Figure 2:**
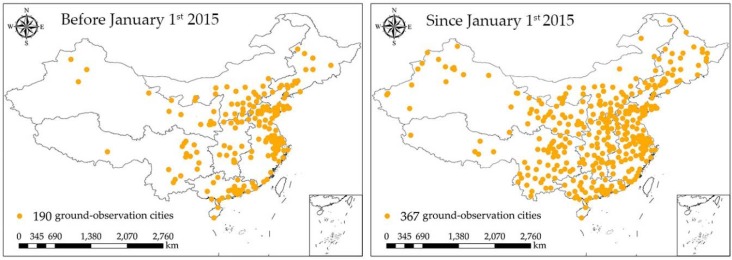
The location of ground-monitoring air quality stations.

The PM_2.5_ and PM_10_ concentrations data used for this study were obtained from website PM25.in (http://pm25.in/about), which collects official real-time air quality data provided by China National Environmental Monitoring Center (CNEMC). The real-time air quality data include hourly PM_2.5_ concentration data (μg/m^3^), hourly PM_10_ concentration data (μg/m^3^), Air Quality Index (AQI), and other airborne pollutants concentration data. Before 1 January 2015, the published PM data supplied by PM25.in (http://pm25.in/about), covered 190 monitoring cities in China, and this number has increased to 367 since 1 January 2015 [30]. The location of ground-monitoring air quality stations can be seen in Figure 2.

By calling the specific API document on website PM25.in (http://pm25.in/about), we collected hourly PM_2.5_ and PM_10_ concentrations data for all monitoring cities in China from 18 January 2013 to 31 December 2017. The daily PM concentration data for each region were calculated by averaging all available hourly PM data from all monitoring cities.

#### 2.2.2. MODIS Active Fire Data

The Moderate Resolution Imaging Spectroradiometer (MODIS) is an optical remote sensing instrument widely used in the fields of Geoscience, Environmental Science, and so on. Owing to its multi-spectral bands (36) and broad spectrum, ranging from 0.4 μm (visible band) to 14.4 μm (thermal infrared band), MODIS can provide a great deal of geographic and atmospheric information. Meanwhile, terra (AM) and aqua (PM) with MODIS transits China four times per day on 10:30, 22:30, 01:30, and 13:30, respectively [31]. Concerning the capability of fire detection, MODIS can monitor conflagration areas over 1000 m^2^. If the weather is suitable (e.g., little/no smoke and relative homogeneous land surface) for observing, one tenth of burning fire spots would be detected. Light fires covering around 50 m^2^ can be detected under the most favorable weather conditions [32].

**Figure 3:**
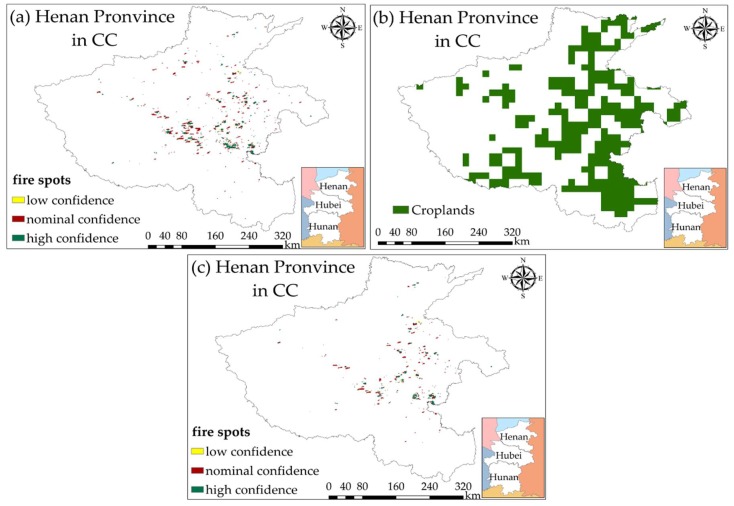
Extraction of crop residue burning spots in China. (**a**) Fire spots extracted from Moderate Resolution Imaging Spectroradiometer (MODIS) fire products; (**b**) Croplands extracted from Land-Use and Land-Cover Change (LUCC) dataset in 2015; (**c**) Crop residue burning spots extracted by combining MODIS fire products and LUCC dataset.

We utilized MOD14A1/MYD14A1 daily Level 3 fire products (MODIS Thermal Anomalies/Fire products) with a spatial resolution of 1 km, which are available at NASA’s LAADS DACC ftp server [33], to extract crop residue burning spots in China. In addition, a contextual algorithm was applied to detect fire spots according to the strong radiation from mid-infrared bands [34]. The products also classified the reliability of fire detection into three levels, including low-confidence fires, nominal-confidence fires, and high-confidence fires. MOD14A1/MYD14A1 were stored as a single file that consisted of eight days’ data for convenience, representing eight-day continuous collection of fire data. To get daily fire spots map (Figure 3a), a maximum value composite method was employed for processing the data integration of MOD14A1/MYD14A1 products.

#### 2.2.3. Land-Use and Land-Cover Data

Although fire spots could be extracted from MODIS fire products, it cannot be directly defined as the crop residue burning spots. Owing to the existence of such burning types as forest fire and urban solid waste incineration, the extraction of crop residue burning spots was further processed with a dataset of Land-Use and Land-Cover Change (LUCC) provided by Resources and Environmental Sciences Data Center, Chinese Academy of Sciences (RESDC) [35]. The dataset reflects changes of land-use and land-cover in China every five years with a high spatio-resolution of 1 km, which is similar to that of MODIS fire products’. This data set has six classes, including cropland, forest, grassland, waters, urban and rural & industrial and residential areas, and unused land. The classification precision of this dataset for each region varies from 73% to 89%, and the overall accuracy of whole nation is up to 81% [36]. In this study, for more reliable extraction of crop residue burning spots, we used the LUCC data in year 2010 and year 2015 (Figure 3b) to generate cropland-masks on study area. Here, the extracted fire spots in year 2013 and 2014 corresponded to cropland-mask in 2010, and fire spots in other years corresponded to cropland-mask in 2015 (Figure 3c).

### 2.3. Methods

Firstly, due to a tremendous amount of pixels comprised, we conducted mosaic processes to compose complete remote sensing images of China. Meanwhile, we extracted “fire-mask” from Science Dataset for obtaining fire spots maps of the study area. Given the long research period and the large quantity of data, we employed batch processing using a specific tool named MODIS Reprojection Tool (MRT) provided by the Land Processes Distributed Active Archive Center. Secondly, in order to summarize overall fire spots in one day, a maximum value composite strategy was proposed and developed to count the number of daily fire spots [18]. The principle of this strategy is to set corresponding attribute values (7 means low-confidence fire spots, 8 means nominal-confidence fire spots, and 9 means high-confidence fire spots) to each pixel based on the maximum value in the daily four observations. In the process of composite, if fire spots detected in the same pixel were recorded several times for a day, we only counted them as one spot to avoid repeat counting. Clouds and haze had significant influences on the detection of fire spots. Since the same area was rarely covered by clouds in the four observations per day, this strategy reduced the occlusion effects and guaranteed the accuracy of fire spots detection. Thirdly, we employed LUCC dataset for extracting crop residue burning spots from the preprocessed data. Cropland-masks were selected from the dataset and combined with corresponding fire spots maps, then daily fire pixels located in croplands (daily crop residue burning spots) were extracted. On the other hand, hourly PM_2.5_ and PM_10_ concentration data were collated into a daily format and the city-level observation data were also recalculated into a regional scale. Finally, we employed statistical and Spearman’s rank correlation analysis to examine the correlation between crop residue burning and PM pollution for each region at different temporal scales.

## 3. Results

**Figure 4:**
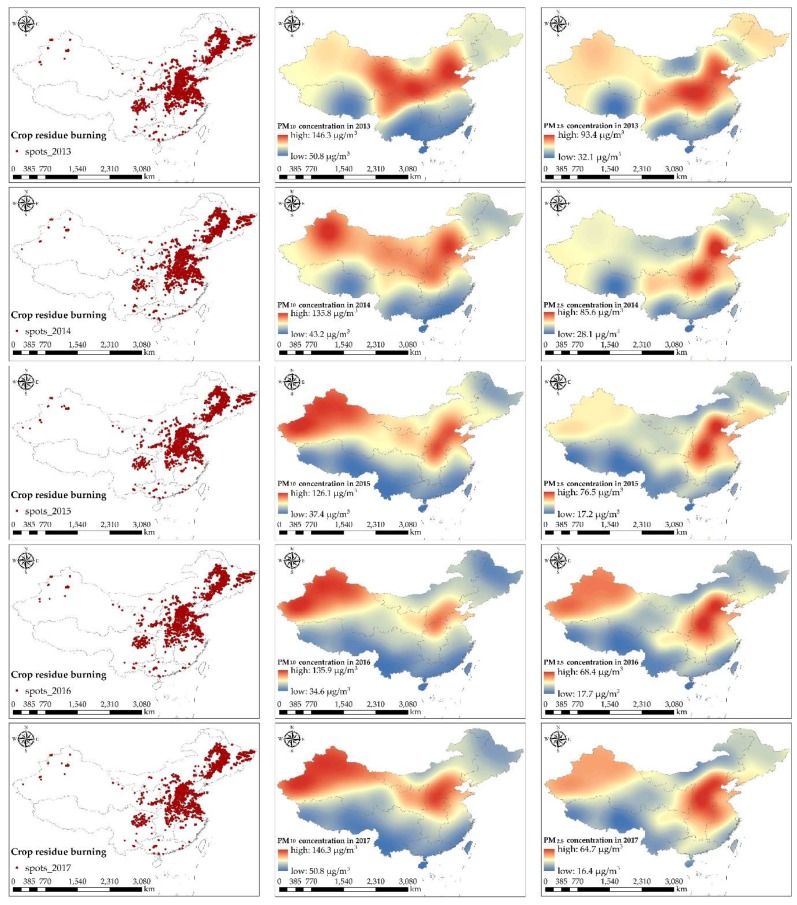
The spatial distribution of crop residue burning and particulate matter (PM) concentrations in the different regions of China. The left column shows the spatial distribution of crop residue burning spots in mainland China. The middle and right columns show the spatial distribution of PM_10_ concentration and PM_2.5_ concentration, respectively, in China by interpolating.

To better understand the following study, the spatial distribution of crop residue burning and PM concentrations in the different regions of China was shown in Figure 4.

### 3.1. The 5-Years’ Variations and Characteristics of PM_2.5_ and PM_10_ in China from a Regional Perspective

#### 3.1.1. Interannual Variations and Characteristics

**Figure 5:**
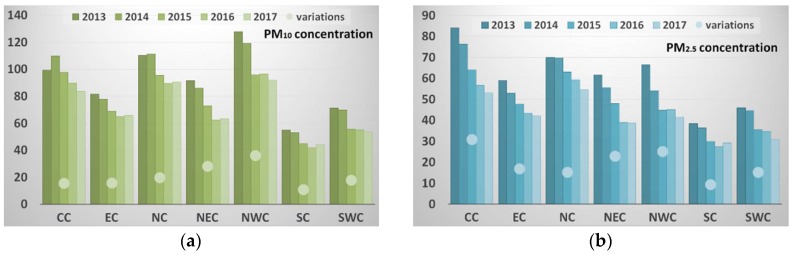
The overall variations of PM_10_ (**a**) and PM_2.5_ (**b**) concentrations in different regions of China from 2013 to 2017. The histogram represents mean PM concentration (μg/m^3^) and the circle refers to the difference between the annual mean PM concentration in 2017 and that in 2013.

**Figure 6:**
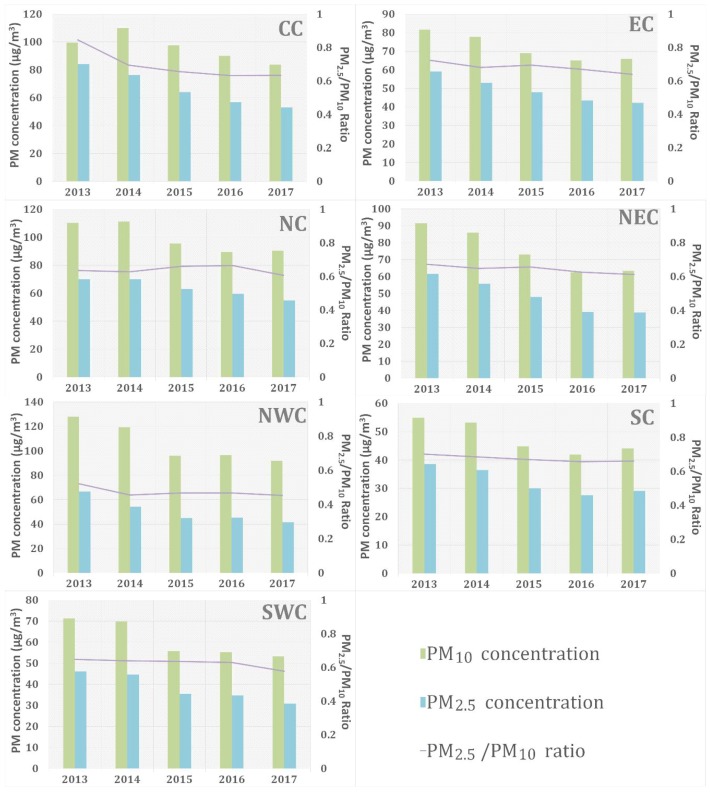
The 5-year variations of PM_2.5_/PM_10_ Ratio and Difference Value (PM_10_–PM_2.5_) in each region of study area.

According to Figure 5, one can see a remarkable downtrend of PM concentrations in all of these seven regions from 2013 to 2017. Specially, during the first three years, PM concentrations in each region decreased dramatically. Afterwards, the decline rate decreased and such regions as SC even demonstrated a slight rise of PM concentrations in 2017. Different from variations of PM_2.5_ concentrations, PM_10_ concentrations from 2016 to 2017 presented a slight upward trend in most regions. The peak value of PM_2.5_ concentrations usually appears in CC and NC. The region with highest PM_10_ concentration is NWC. Similarly, a clear decline of PM_2.5_ concentrations and PM_10_ concentrations was witnessed in CC and NWC, respectively. The decrease of PM concentrations in NEC was relatively higher than that of other regions. Furthermore, we analyzed the PM_2.5_/PM_10_ ratio, which could reveal different characteristics and origins of particle pollution [36]. A higher ratio usually indicated that PM pollution was caused by anthropogenic activities, while a lower ratio demonstrated that natural factors were the main contribution source of PM pollution [37]. According to Figure 6, the PM_2.5_/PM_10_ ratio in each region all dropped to a much lower level with small fluctuations that occasionally arose during 5-year period. Meanwhile, the most obvious decline of PM_2.5_/PM_10_ ratio was shown in CC (from 0.85 in 2013 to 0.63 in 2017) and the lowest ratio appeared in NWC (average value is about 0.47) for each year.

#### 3.1.2. Seasonal Variations and Characteristics

**Figure 7:**
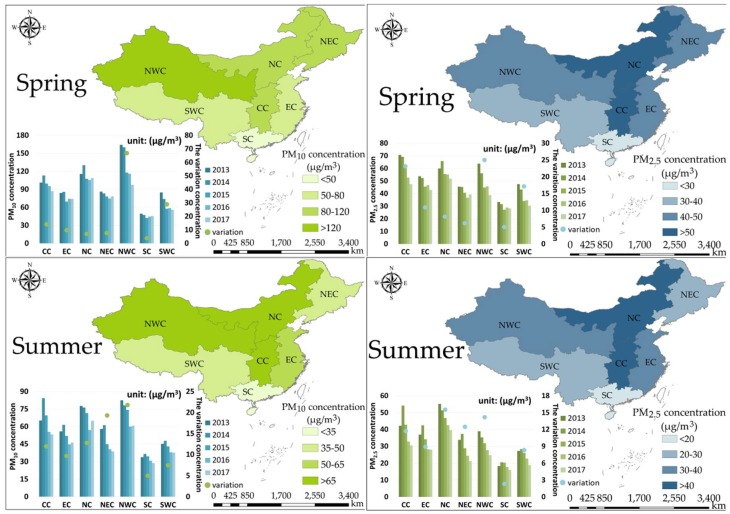

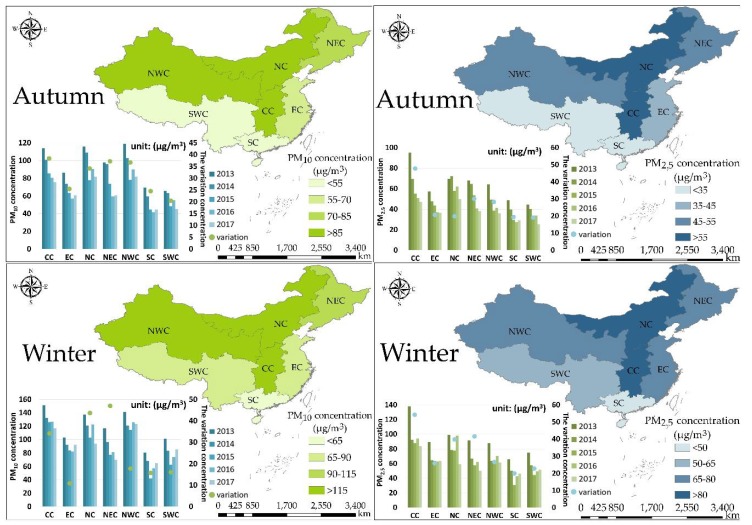
The characteristics and variations of PM_10_ and PM_2.5_ concentrations in different regions of China from seasonal and interannual perspectives.

For better understanding seasonal variations and characteristics of PM_2.5_ and PM_10_ concentrations, we divided twelve months into four seasons as follows: Spring (March, April, May), summer (June, July, August), autumn (September, October, November), and winter (December, January, February). As can be seen from Figure 7, the seasonal variation of PM_10_ concentrations in the same region is similar to that of PM_2.5_ concentrations, whereas seasonal characteristics and variations of these two PM concentrations vary significantly across regions. Besides, concentrations of PM_10_ and PM_2.5_ in each region both demonstrated a generally decreasing tendency in each season, despite some obvious concentration-growth in such years as 2014 and 2016.

Regarding characteristics of PM_10_ concentrations in different regions, the highest value always appeared in NWC and the lowest concentration of PM_10_ was usually observed in SC. In addition, throughout a whole year, the average PM_10_ concentration of NC always maintained a much higher level than that of other regions’. For CC and NEC, the PM_10_ pollution usually deteriorated in autumn and winter. Moreover, from a temporal perspective, the maxima of PM_10_ concentrations in each region appeared in winter, and the minima appeared in summer. In spring, PM_10_ concentrations evidently decreased in NWC and slightly decreased in other regions. For the decline of PM_10_ concentration in summer, the maximum change appeared in NWC, with NEC in the second place. In autumn, the declines from 2013 to 2015 were evident in all regions and increases appeared in northern and western China in 2016, when PM_10_ concentrations in CC, NEC, and NWC greatly reduced (40 μg/m^3^ approximately) compared to the previous high concentration. For winter, the major decrease of PM_10_ concentrations was witnessed in NEC, NC, and CC.

Similar to PM_10_ concentrations, PM_2.5_ concentrations in different regions were the lowest in summer and highest in winter. Spatially, the peak of PM_2.5_ concentrations usually appeared in CC and NC, which was different from that of PM_10_ concentrations. Meanwhile, the lowest PM_2.5_ concentration showed in SC, which was similar to that of PM_10_ concentrations. For other regions, the PM_2.5_ concentration of NEC always kept at a much higher level in spring, autumn, and winter. Although the PM_2.5_ concentration of NWC was not the highest in these seven regions, it remained at a relatively high level throughout the year. The higher PM_2.5_ concentration was also observed in EC in spring, summer, and winter. PM_2.5_ concentration in SWC was lower than other regions except for SC. For spring, the notable decline of PM_2.5_ concentrations was witnessed in NWC and CC, whilst the decrease in other regions was much smaller. For summer, the decline of PM_2.5_ concentrations was very small in each region and the largest decrease of 16 μg/m^3^ appeared in NC. Different from slight variations in spring and summer, PM_2.5_ concentrations in autumn and winter decreased significantly in each region. Particularly, maximum changes were observed in CC (reduced about 50 μg/m^3^) and NEC (reduced about 35 μg/m^3^). Besides, for NC, the decreased-concentration in winter was much higher than that in autumn. Other seasonal-interannual variations of PM concentrations could be found in Figure 7.

### 3.2. The 5-Year Variations of Crop Residue Burning in China from Regional Perspective

#### 3.2.1. Interannual Variations

**Figure 8:**
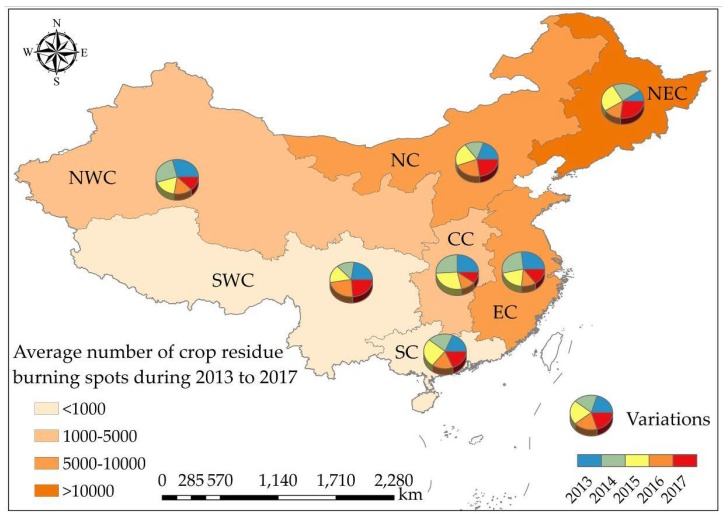
Interannual variations of crop residue burning spots in different regions of study area.

According to Figure 8, the most serious region of crop residue burning was NEC, with an annual average number of crop residue burning spots up to 30,569 during the five years period. Meanwhile, throughout China the number of crop residue burning spots progressively reduced from east to west. Specifically, the decline of burning spots in NWC and EC was the most obvious without large fluctuations. The number of crop residue burning spots in CC decreased significantly in the past five years, whereas during the first three years, the number actually increased gradually until 2016, when a significant decrease showed up. The number of crop residue burning spots in NEC increased significantly from 2014 to 2015. Although the number dropped to a relatively low level in 2016, it rose in 2017 to three times of the number in 2013. Similarly, the number of crop residue burning spots in NC also increased generally, except for the decrease in 2014. Compared with the north of China, the number of crop residue burning spots distributed in SC and SWC were small and interannual variations of burning spots in these two regions were very slight.

#### 3.2.2. Seasonal Variations

**Figure 9:**
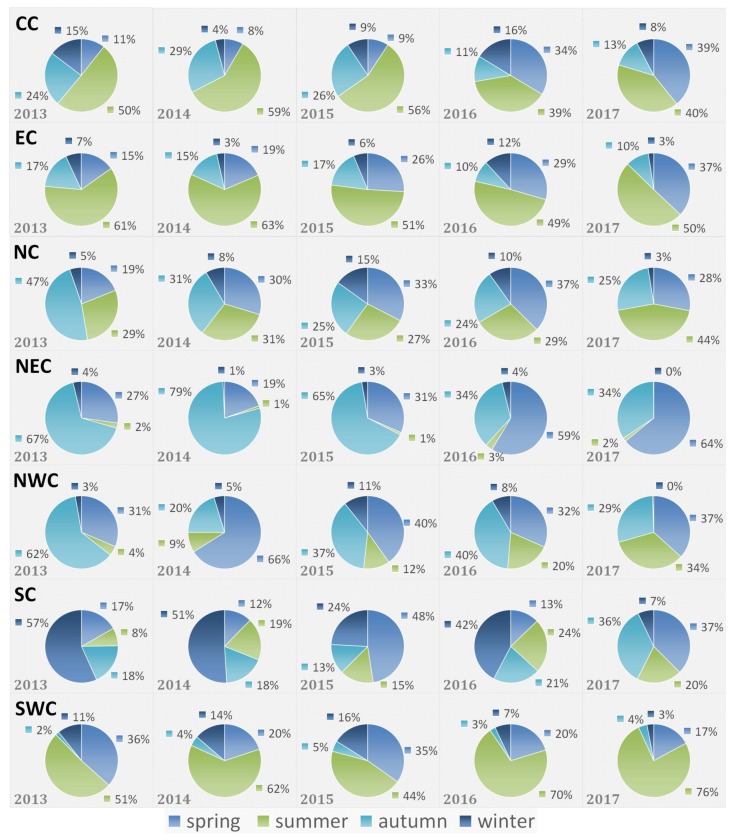
Seasonal variations of crop residue burning spots in different regions of study area.

According to Figure 9, we can see clear seasonal variations of crop residue burning spots for each region. Crop residue burning in CC usually took place in summer and autumn. During 2013 to 2017, the proportion of crop residue burning in spring increased gradually, and decreased notably in summer and autumn, whilst it demonstrated slight variations in winter. The variation of crop residue burning in EC were generally consistent with that in CC. For NC, crop residues were often burnt in summer and autumn. However, the proportion of crop residue burning spots in these two seasons decreased year by year, while the ratio in spring gradually increased to one third of the total amount. The number of crop residue burning spots were limitedly distributed in winter. As an agriculturally developed region, NEC experienced very intense crop residue burning, which mainly concentrated in spring and autumn. Meanwhile, the proportion of crop residue burning in autumn decreased from 67% in 2013 to 34% in 2017, and the proportion in spring increased from 27% in 2013 to 64% in 2017. For NWC, crop residue burning mainly took place in spring and autumn. A sudden increase appeared in the spring of 2014, whilst the proportion in autumn plummeted to 20%. Following this, crop residue burning in spring and autumn decreased dramatically, and gradually concentrated in summer. During this period, the proportion of crop residue burning in autumn decreased whilst the proportion in spring stabilized between 30% and 40%. Finally, crop residue burning spots in NWC presented similar proportion in spring, summer and winter in 2017. Unlike the northern part of China, crop residue burning in SC was usually observed in winter. Whereas, in recent years, proportions of crop residue burning in other seasons increased without clear pattern. Furthermore, crop residue burning of SWC usually concentrated in spring and summer. During this period, the proportion of crop residue burning increased in summer and decreased in spring.

### 3.3. The Correlation between PM Concentration and Crop Residue Burning at Different Temporal Scales

#### 3.3.1. The Correlation between PM Concentrations and Crop Residue Burning at an Annual Scale

**Figure 10:**
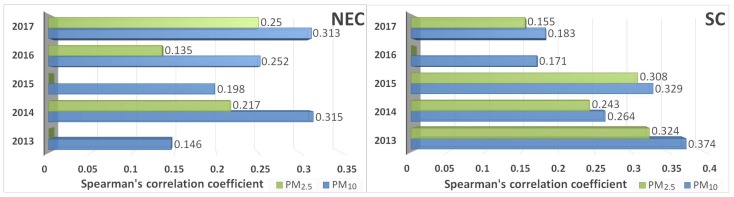
Interannual variations of correlation coefficient between PM concentrations and crop residue burning in Northeast China (NEC) and South China (SC).

We employed Spearman’s rank correlation for establishing the correlation between daily PM data and daily crop residue burning spots data. The result (Table 1) showed that the correlation between PM concentration and crop residue burning in NEC and SC were much stronger than that in other regions. According to Figure 10, variations were different in these two regions. In NEC, correlations between PM_10_ concentration and crop residue burning were generally upward with fluctuations, except for a notable decrease in 2015. The overall trend of the correlation between crop residue burning and PM_2.5_ concentrations was similar, yet the significance of this correlation was much weaker. In SC, correlation coefficients between PM concentrations and crop residue burning generally decreased, except for a slight increase in 2015. In addition, a significant phenomenon was that the correlation between PM_10_ concentrations and crop residue burning was stronger than that between PM_2.5_ concentrations and crop residue burning.

#### 3.3.2. The Correlation between PM Concentrations and Crop Residue Burning at a Seasonal Scale

**Figure 11:**
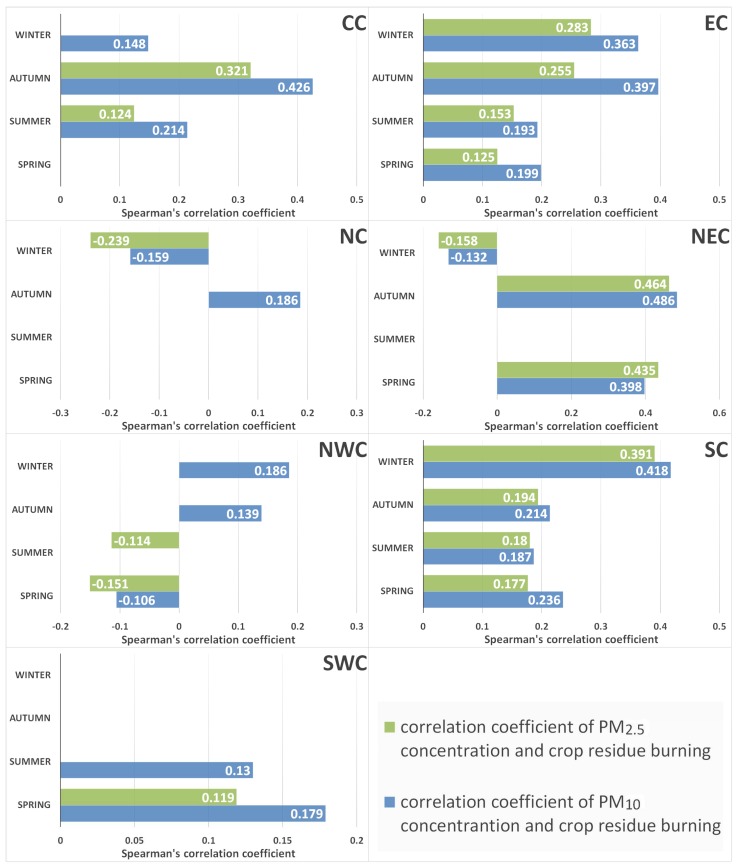
Seasonal variations of correlation coefficient between PM concentrations and crop residue burning among seven regions of China.

We analyzed correlations between PM concentrations and crop residue burning for each region from a seasonal perspective. The results (in Figure 11 and Table 2) showed that correlations in autumn were significantly stronger for the north part of China, including CC, EC, and NEC. For SC, correlations were stronger throughout four seasons and the largest correlation coefficient appeared in winter. Correlations in SWC were relatively poor and only significant in spring and summer. The correlation coefficient in NEC was the strongest among seven regions and the strongest correlation usually appeared in spring and autumn, when crop residues were intensely burnt in NEC. For EC, the correlation between PM concentrations and crop residue burning was significant in four seasons and were much stronger in autumn and winter. Similar to annual analysis, PM_10_ concentrations were more strongly correlated with crop residue burning than PM_2.5_ concentrations.

#### 3.3.3. The Correlation between PM Concentrations and Crop Residue Burning in Burning-Concentrated Periods

With different time of crop ripening in each region, periods of crop residue burning are different accordingly. Therefore, in order to better analyze the change of PM concentrations when crop residues were intensely combusted, for each year, we selected a burning-concentrated period for each region during 2013–2017. The principle of selection was based on the appearance of peak months of crop residue burning spots and prior knowledge of agricultural production. In total, we acquired five periods for each region and analyzed the correlation between the number of crop residue burning spots during the burning-concentrated period and corresponding PM_2.5_ concentrations. The results are shown in Table 3. Except for NC, correlations between PM concentrations and crop residue burning were significant in all regions. Generally, correlations in NC and SWC were the weakest, and correlations in NEC were the strongest. Meanwhile, the correlation between PM_10_ concentrations and crop residue burning was significantly stronger than that of PM_2.5_ concentrations. This result indicated that the variation of PM_10_ concentrations was more sensitive to crop residue burning than that of PM_2.5_ concentrations during the process of crop residue burning. Correlation between PM concentrations and crop residue burning increased significantly with the narrowing temporal scales and was the strongest during burning-concentrated periods, indicating that intense crop residue burning exerts a much stronger influence on the short-term than long-term variation of PM concentrations.

## 4. Discussion

### 4.1. The Attribution of Variations of PM_10_ and PM_2.5_ Concentrations during 5-Year Period

In this study, we analyzed variations and characteristics of PM concentrations from interannual and seasonal perspectives. Meanwhile, we selected some crop residue burning-concentrated periods to explore variations of PM concentrations during the burning processes. Generally, concentrations of PM_10_ and PM_2.5_ have decreased notably since 2013. Besides, PM_2.5_/PM_10_ ratios also declined during the 5-year period which indicates that the composition of PM_10_ occupied by PM_2.5_ is decreasing. Meanwhile, some studies have shown that the high PM_2.5_/PM_10_ ratio can be attributed to human activities, while the lower ratio is related to natural factors [37,38]. In other words, PM_2.5_ pollution has been mitigated significantly, due to a series of emission-reduction measures. Firstly, in autumn and winter, the variation of PM concentrations in northern China can be attributed to the control of crop residue burning, traffic exhaust, and coal combustion for large-scale central heating [39]. Secondly, with the implementation of Red and Orange alert measures for reducing PM pollution, PM_2.5_ concentrations have decreased remarkably [40]. Thirdly, as a result of traffic control, the exhaust-emission of vehicles has been cut down dramatically and leads to the reduction of PM concentrations [41]. Fourthly, some environmental-meteorological projects have been implemented to address PM pollution issues [42]. In burning-concentrated periods, the variation trend of PM concentrations is consistent with that of crop residue burning in all regions, indicating intensive crop residue burning leads to instant deterioration of PM concentrations. Hence, more strict and effective policies should be proposed and implemented to encourage more efficient utility of crop residues and reduce large scale and intensive crop residue burning.

### 4.2. The Attribution of Correlations between PM Concentration and Crop Residue Burning

The correlation between PM concentrations and crop residue burning was discussed in this paper. Firstly, it is found that the correlation between PM concentrations and crop residue burning is significant and strong, especially in burning-concentrated periods, which is consistent with findings from previous studies [43]. Awasthi et al. (2010) found PM_10_, PM_2.5_, PM_10–2.5_ concentrations increased significantly during crop residue burning in India. Strong correlation between crop residue burning and PM concentrations was observed. Different from this research, Awasthi et al. (2010) found that the PM_2.5_ concentration was more sensitive to crop residue burning than PM_10_ concentrations. This difference may result from pollution level and meteorological diffusion conditions in India. However, our finding about the very strong correlation between crop residue burning and PM concentrations during the intensive crop residue burning period in all regions across China proved that, despite other influencing factors such as emission sources and meteorological factors, intensive and large-scale crop residue burning could be a dominant emission sources for PM pollution across China. Secondly, correlations between different particulate matters and crop residue burning are distinct. PM_10_ concentrations are much strongly correlated with crop residue burning than PM_2.5_ concentrations, indicating crop residue burning in China may produce more PM_10_ than PM_2.5_. From a temporal perspective, crop residue burning in autumn usually presents a higher correlation with PM concentration, which is consistent with the findings from Yin et al. Whereas, different from this research, Yin et al.’s research [23] mainly introduced the temporal variation of both crop residue burning and PM_2.5_ concentrations in China and did not discuss the correlation from different temporal scales. From a spatial perspective, the correlation in NEC is the strongest among the seven regions, especially in spring and autumn, suggesting that the PM concentration is closely related to crop residue burning in the burning-concentrated periods. This phenomenon was consistent with findings from previous studies suggesting that crop residue burning is related to PM_2.5_ concentration [23,24]. The main reason for the poor correlation in NC is that the source of PM is high exhaust-emission of vehicles and industrial production, instead of crop residue burning [41]. For NWC, petroleum exploitation is also an important contributor to PM pollution [44], which may be the reason why PM_10_ demonstrates a weaker correlation with crop residue burning than PM_2.5_. To sum up, the burning of crop residues has a great contribution to PM pollution, though the relative contribution of crop residue burning to PM concentrations, compared with other emission sources, including industry and traffic exhaust, should be further investigated.

### 4.3. Limitations and Prospect

Although the paper comprehensively examined correlations between PM concentration and crop residue burning, some limitations remain. Firstly, due to the fact that crop residue burning usually lasts for a short period, the correlation analysis should be more reliable if it is conducted based on a finer temporal resolution, such as hourly. Thus, considering the finer temporal resolution of Himawari-8, it is a better choice to extract fire spots on the hourly scale. Secondly, due to the limited spatial resolution of MODIS data, some actual burning spots may be lost in the process of fire spots extraction and statistics. That means remote sensing data with higher temporal resolution are required for extracting fine-scale crop reside burning spots. Furthermore, due to complicated interactions between PM and meteorological factors, commonly used correlation analysis may be biased significantly. To reduce the influence from other factors and better investigate the influence of crop residue burning on PM concentrations, advanced causality methods, such as cross convergent mapping (CCM) [45] and chemical transport models (CTM), such as WRF-CAMx [46], should be employed in future studies. Whereas, the difficulty for examining the causality of crop residue burning on PM concentration without other influencing factors, using above models lies in the short time series of the concentrated crop residue burning periods. Meanwhile, the MODIS data extracted crop residue burning spots are mainly based on a daily scale and thus the time series of intensive crop residue burning is limited to less than 30 numbers, not sufficient for a robust CCM or CTM analysis. Therefore, to implement CCM or CTM analysis, fire spots should be extracted using remote sensing data with a much higher temporal resolution, such as Himawari 8 with 10-min temporal resolution. In the future, with growing availability and accuracy of Himawari data sources, it is possible to conduct robust causality analysis based on CCM or CTM using long time series data of crop residue burning and PM pollution. In this case, the influence of crop residue burning on PM concentrations can be better extracted by filtering the biases of other influencing factors. 

## 5. Conclusions

This paper analyzed interannual and seasonal variations of PM_10_ and PM_2.5_ concentrations and simultaneous variations of crop residue burning in several regions across China. The results showed that the PM concentration was in a downward trend from interannual and seasonal perspectives and PM_2.5_/PM_10_ ratios in different regions decreased gradually. The peak value of PM_10_ concentrations usually appeared in NWC and winter whilst the peak value of PM_2.5_ concentrations appeared in NC and CC. Temporal variations of PM_2.5_ are similar to that of PM_10_ concentrations. For the number of crop residue burning spots in China, it remained a downward tendency during the 5-year period in most regions, except for an evident increase in NEC in 2017. Furthermore, we analyzed correlations between PM concentration and crop residue burning and explored at different temporal scales. The variation of PM_10_ concentration was more sensitive to crop residue burning than that of PM_2.5_ concentrations and the strongest correlation between PM concentrations and crop residue burning appears in NEC. Correlation between PM concentrations and crop residue burning increased significantly with the narrowing temporal scales and was the strongest during burning-concentrated periods, indicating that intense crop residue burning exert a much stronger influence on the short-term than long-term variation of PM concentrations. The methodology and conclusions from this study provide useful reference for better understanding the influence of crop residue burning on PM concentrations at different scales and suggest that intensive crop residue burning leads to instant increases of PM concentrations. Given the major contribution of crop residue burning to PM pollution, more strict and effective policies should be proposed and implemented to encourage more efficient utility of crop residues and reduce large scale and intensive crop residue burning.

## Figures and Tables

**Table 1:** The correlation between particulate matter (PM) concentrations and crop residue burning occurred in different regions of China during 2013 to 2017.

		CC	EC	NC	NEC	NWC	SC	SWC
Spearman	PM10	0.095 **	0.110 **	−0.011	0.218 **	−0.027	0.260 **	−0.019
	PM2.5	−0.015	0.002	−0.106 **	0.124 **	−0.134 **	0.228 **	−0.068 **

Note: ** *p* < 0.01.

**Table 2:** The seasonal variation of correlation coefficients in different regions from 2013 to 2017.

		Spring	Summer	Autumn	Winter
CC	PM_10_	0.063	0.214 **	0.426 **	0.148 **
	PM_2.5_	−0.056	0.124 **	0.321 **	0.003
EC	PM_10_	0.199 **	0.193 **	0.397 **	0.363 **
	PM_2.5_	0.125 **	0.153 **	0.255 **	0.283 **
NC	PM_10_	0.019	0.088	0.186 **	−0.159 **
	PM_2.5_	0.035	−0.009	0.040	−0.239 **
NEC	PM_10_	0.398 **	0.032	0.486 **	−0.132 **
	PM_2.5_	0.435 **	−0.060	0.464 **	−0.158 **
NWC	PM_10_	−0.106 *	−0.013	0.139 **	0.186 **
	PM_2.5_	−0.151 **	−0.114 *	0.087	0.007
SC	PM_10_	0.236 **	0.187 **	0.214 **	0.418 **
	PM_2.5_	0.177 **	0.180 **	0.194 **	0.391 **
SWC	PM_10_	0.179 **	0.130 **	0.068	0.042
	PM_2.5_	0.119 *	0.023	0.063	0.091

Note: * *p* < 0.05; ** *p* < 0.01.

**Table 3:** The correlation between PM concentrations and crop residue burning occurred in different regions of China during burning-concentrated periods.

		CC	EC	NC	NEC	NWC	SC	SWC
Spearman	PM_10_	0.362 **	0.444 **	0.236 **	0.491 **	0.347 **	0.436 **	0.234 **
	PM_2.5_	0.335 **	0.404 **	0.044	0.446 **	0.407 **	0.400 **	0.169 *

Note: * *p* < 0.05; ** *p* < 0.01.
